# Impact of NICE technology appraisal guidance TA566 on access to cochlear implantation for children using hearing aids in the United Kingdom: a multisite observational study

**DOI:** 10.1136/archdischild-2023-326055

**Published:** 2024-05-23

**Authors:** Catherine F Killan, Derek J Hoare, Douglas E H Hartley, Paige Church, Fay Isherwood, Sophia Nasim, Jason Cropper, Robert Gardner, Karen Willis, Nathan Waite, Lynette Garnett, Sara Morgan, Padraig T Kitterick

**Affiliations:** 1 Bradford Teaching Hospitals NHS Foundation Trust, Bradford, UK; 2 National Institute for Health and Care Research (NIHR) Nottingham Biomedical Research Centre, Hearing Sciences, Mental Health and Clinical Neurosciences, School of Medicine, University of Nottingham, Nottingham, UK; 3 Nottingham University Hospitals NHS Foundation Trust, Nottingham, UK; 4 Calderdale and Huddersfield NHS Foundation Trust, Huddersfield, UK; 5 National Acoustic Laboratories, Sydney, New South Wales, Australia

**Keywords:** Audiology, Deafness, Rehabilitation, Technology

Early intervention is essential for deaf and hard-of-hearing children’s oral/aural language development. For sensorineural hearing loss, devices include acoustic hearing aids (HAs) and cochlear implants (CIs). Access to CIs in the UK is subject to technology appraisal (TA). The TA audiometric criterion defines which children are referred from HA to CI services for candidacy assessment and influences candidacy. TA166 required unaided air-conduction hearing threshold levels (HTLs) of ≥90 dB HL at 2 kHz and 4 kHz bilaterally.^1^ TA566 expanded this to at least two HTLs of ≥80 dB HL bilaterally, including 0.5, 1, 2, 3 and 4 kHz.^2^

We assessed how TA566 changed children’s eligibility for CI referral using four hospitals’ paediatric HA caseloads (Bradford, Huddersfield, Calderdale and Nottingham). Non-identifiable routine audiology data were collected during 2019–2021. Included cases were <18 years old and prescribed at least one HA but no CI. Bone conduction aid (BCA) users were excluded as BCA and CI indications did not overlap. Whole caseloads were screened to avoid selection bias. Ascertainment was reported to be 100%, although this could not be verified. Device(s) prescribed and behavioural or electrophysiological HTLs (bilateral ear-specific or binaural soundfield) were extracted. Children were classified as falling within, outside or being unclassifiable by TA166^1^ and TA566.^2^ Numbers of children not eligible, newly eligible, of unknown eligibility and previously eligible for CI referral were derived and the percentage of HTLs measured at each frequency were calculated for each group.

**Figure 1:**
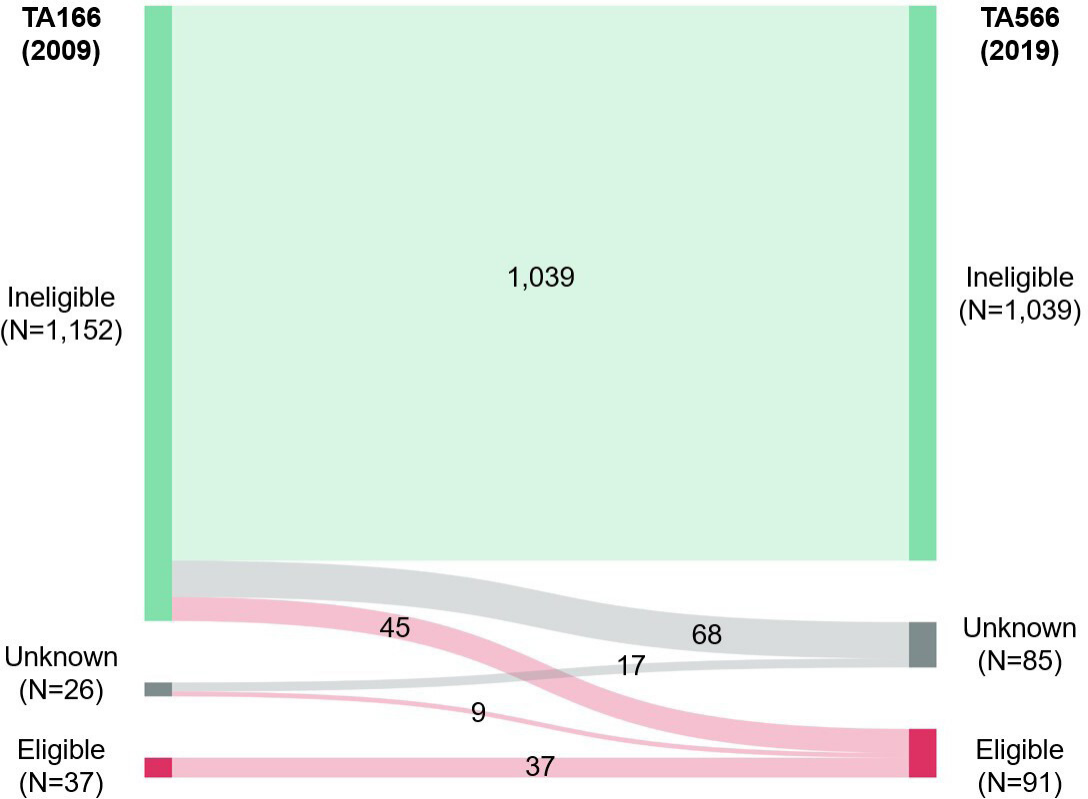
Sankey diagram of the impact of TAG566 on children’s audiometric eligibility for cochlear implantation. For both the 2009 (left axis, National Institute for Health and Care Excellence Technology Appraisal Guidance 166) and 2019 (right axis, National Institute for Health and Care Excellence Technology Appraisal Guidance 566) guidance. Children whose unaided hearing thresholds from the two most-recent audiograms made them audiometrically ineligible, of unknown eligibility, or eligible are represented by green, grey and red vertical bars respectively. The numbers in the centre of the figure describe the number of children whose status did or did not change from and to each category. TA, technology appraisal.

Figure 1 shows changes in audiometric eligibility.

**Figure 2:**
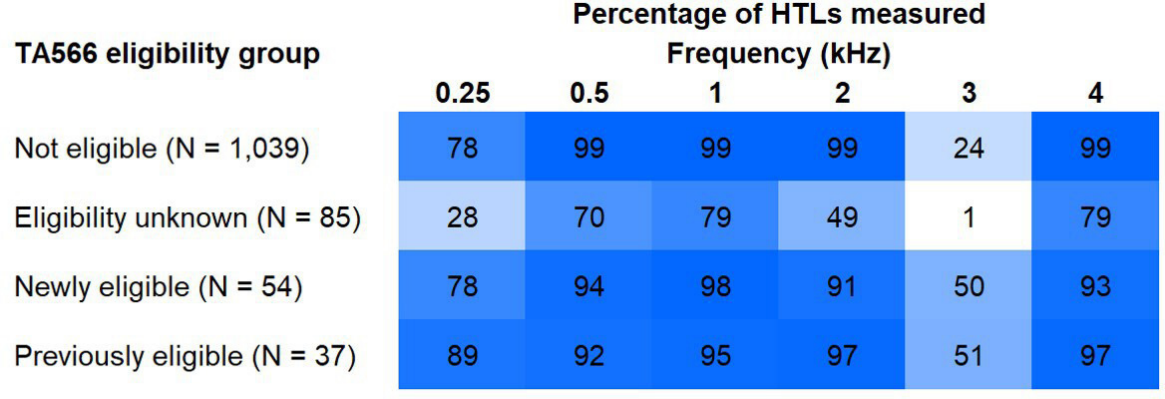
Heat map of proportion of unaided hearing thresholds recorded on the two most-recent audiograms for each audiometric frequency by TA566 eligibility group. HTL, hearing threshold level; TA, technology appraisal.

Figure 2 shows the percentage of cases for which HTLs at each frequency were available, by audiometric eligibility group. Data are for right ears where ear-specific HTLs were available and binaural soundfield thresholds otherwise.

TA566 expanded the proportion of children audiometrically eligible for CI referral from 3.0% to 7.5%. However, the rate of new child CI recipients UK-wide, which had been rising, plateaued following TA566’s implementation in June 2019 then fell and stabilised below prepandemic levels.^3^ Research is needed to determine whether this was due to reduced incidence or detection of hearing loss, fewer referrals offered, parents declining referrals, referred children not being offered CIs or other causes.

TA566 increased the proportion of children whose audiometric eligibility for CI referral was unknown, from 2.1% to 7.0%. This was due to missing HTLs across frequencies, most markedly at 3 kHz. Full audiograms should be measured where this influences management. To promote equitable access to CIs for children less able to supply full audiograms due to, for example, age or developmental delay, research could establish referral and/or candidacy guidance requiring fewer HTLs.

The caseloads sampled may not have been representative of all children using HAs in the UK. Conductive overlays and non-audiometric candidacy criteria assessed by CI services, including language development and fitness for surgery, could not be considered. Teenagers <18 years old on adult caseloads were not captured. Nonetheless, this study provides a benchmark against which HA and CI services can compare referral rates and will inform future audiometry practice and referral guidance.
